# Contextual Determinants of General Household Hygiene Conditions in Rural Indonesia

**DOI:** 10.3390/ijerph182111064

**Published:** 2021-10-21

**Authors:** D. Daniel

**Affiliations:** 1 Department of Water Management, Faculty of Civil Engineering and Geosciences, Delft University of Technology, 2628 CN Delft, The Netherlands; d.daniel@tudelft.nl; 2 Department of Health Behaviour, Environment, and Social Medicine, Faculty of Medicine, Public Health and Nursing, Universitas Gadjah Mada, Yogyakarta 55281, Indonesia

**Keywords:** hygiene, sanitary inspection, rural areas, Indonesia

## Abstract

Household hygiene is critical to prevent pathogen transmission at the household level. Assessing household hygiene conditions and their determinants are needed to improve hygiene conditions, especially in rural and less developed areas where the housing conditions are relatively worse than they are in urban areas. This study used data from 278 household interviews and observations in rural areas in the district of East Sumba, province East Nusa Tenggara, Indonesia. The data were analyzed using statistical methods. In general, the household hygiene conditions in the study need to be improved. The main potential sources of pathogen transmission were from the surrounding environment, i.e., non-permanent floor and garbage, and personal hygiene, i.e., handwashing facilities with water and soap were only observed in the homes of four out of ten respondents. The presence of livestock roaming freely in the house’s yard was another source of contamination. Easy access to water and wealth significantly influenced the hygiene conditions. Implementing low-cost interventions, i.e., cleaning the house of garbage and animal feces and cleaning nails, should be the priority in immediate intervention, while providing easier access to water supply, especially during the dry season, could be a long-term intervention. This paper also argues that analyzing household hygiene conditions or practices should be complemented by analyzing contextual determinants of the hygiene conditions or practices, so that we can develop more precise intervention by considering the local or household context.

## 1. Introduction

Appropriate water, sanitation, and hygiene (WASH) practices can prevent the transmission of various infectious diseases. Empirical evidence indicates that household water treatment and safe storage (HWTS) reduces the prevalence of diarrhea among children [1]. Handwashing can reduce cases of diarrhea [2]. Furthermore, despite variations in the results of the effect of proper sanitation practices to reduce diarrhea [3], there is a consensus among scholars that safe excreta disposal can provide a barrier to fecal contamination in the household [4].

Since pathogens can reach humans by various routes, a holistic approach or multi-barrier prevention to minimize the risk of pathogens reaching a human is suggested [5,6]. Multi-barrier prevention has been applied in the context of the drinking water supply, i.e., the water safety plan [7]. This approach first starts by identifying potential sources of contamination and then develops relevant interventions to minimize or eliminate the contamination sources. In the context of pathogen transmission in a house, an article by Ercumen et al. [8], in their introduction, elaborates nicely on how water, sanitation, and hygiene practices complement each other to provide multi-barrier prevention for pathogen transmission in a house. For example, they argue that coupling HWTS and handwashing can prevent the recontamination of treated water by dirty hands.

In identifying potential sources of contamination, one can use and adapt the sanitary inspection (SI) form. The SI form consists of questions on potential sources of contamination in specific settings, e.g., pipe distribution network, water sources, or household drinking water [9]. The SI was first introduced by the World Health Organization in 1997 [10]. The SI has been widely used, especially in drinking water safety programs [9]. Daniel et al. [11] suggest adapting the SI forms to “the local context to maximize their applicability”. Since there is no “standard” SI available for measuring general household hygiene conditions, the variables that are measured can be inspired by previous studies or available and relevant SI forms. There are some studies on household hygiene conditions that can inspire the questions used to measure household hygiene conditions, e.g., by [12,13]. Questions or variables from the SI form for household drinking water can also be used since they measure hygiene practices related to drinking water; see, for example, the studies of [5,11,14]. One can then use the adapted SI forms to inform which hygiene aspects should be tackled, which can then improve the general household hygiene conditions.

Some hygiene-related studies have been conducted in Indonesia, for example, on handwashing practices [15,16,17] and general household hygiene conditions [5,13]. There are is also evidence that household hygiene is associated with children’s malnutrition in Indonesia [18,19]. Furthermore, children living the in rural areas of Indonesia were more likely to be stunted compared to children in urban areas [20,21]. Other studies found that disparities in the WASH conditions or facilities between urban and rural areas exist in Indonesia [22,23,24]. 

However, studies on the determinants of general household hygiene conditions in rural Indonesia are lacking. This study then aims to fill that gap and aims to investigate the hygiene conditions in households in rural Indonesia. Furthermore, we seek to find contextual determinants, i.e., socio-economic or socio-environmental determinants, of hygiene conditions. These are believed to influence health-related behavior [25,26], including household hygiene practices or conditions. This study provides a snapshot of the household hygiene conditions in less developed areas and indigenous communities in developing countries that are vulnerable to WASH-related diseases and insecurities, whereas reaching those left behind is the key to achieve Sustainable Development Goals 6.1 and 6.2 by 2030 [27]. Furthermore, this study may inspire potential interventions to improve the hygiene conditions of rural households, especially in Indonesia, where there are still many less developed, remote, and also indigenous communities. Finally, this study also shows how more specific interventions can be provided by considering the data of household hygiene conditions and contextual determinants, i.e., socio-economic conditions of the households or community.

## 2. Materials and Methods

### 2.1. Data Collection

**Figure 1:**
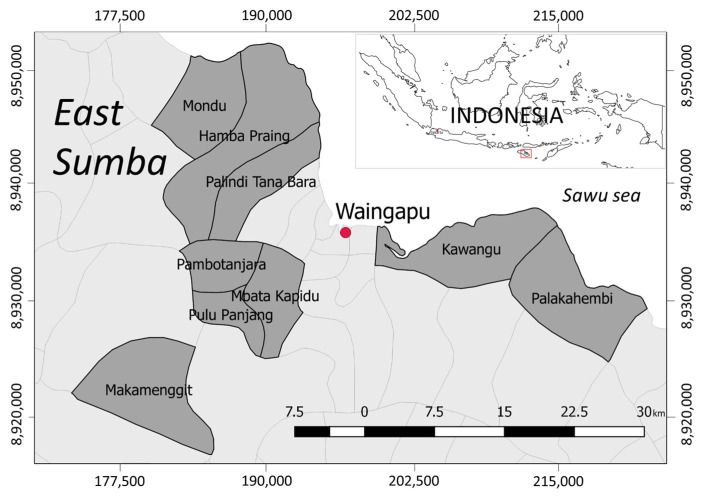
Location of the study area in the district of East Sumba.

A cross-sectional study was conducted in the district of East Sumba, Province East Nusa Tenggara, Indonesia, in July–August 2019 (Figure 1). According to the district data, about 30% of the total population practiced open defecation in 2017 [28]. A total of 40% of the total households in this district used wells as their main water source, and only 18% had access to tap water in 2017 [29]. The tap water is distributed without treatment. Furthermore, severe drought usually occurs in April–October, resulting in many people, especially those in the rural parts of the region, face serious water supply problems [30]. 

An indigenous belief, called “Marapu”, is commonly practiced by people in the study area and has a large influence on the daily life of the people [31]. Moreover, poverty is a serious problem in this area, and the prevalence of child malnutrition is also high [32,33]. About 30% of the total population in that district were categorized as “poor households”, i.e., cannot afford basic food. The level of school drop-out is also high, i.e., more than 20% [34]. 

For this study, 328 households in 9 villages were visited. This visit in 2019 is the continuation of a previous WASH study in 2018. In 2018, the households that were visited were randomly selected during a transect walk within nine villages and enrollment was asked of every, for example, five houses. Information on village selection has been discussed in a previous study [35,36]. Data collection was conducted by six local enumerators who were trained to conduct a household interview, sanitary inspection, and water sampling. 

An article by Sonego and Mosler [12] indicated data collection approaches to study hygiene practice: self-reports, structured observations, and spot-checks. Moreover, Kelly et al. [9] also suggest complementing SI with water quality analysis, especially in the context of water safety. Therefore, in this study, the water quality data were included in the analysis. The drinking water samples were collected from all respondents, and we assessed the presence or absence (P/A) of fecal contamination in the drinking water storage, i.e., presence of *E. coli* in a 1 mL water sample. More information about the sampling procedure and analysis can be found in another study [5]. The observational data were collected using the Open Data Kit (ODK) software on a smartphone and were then transferred to a computer for analysis. 

This study protocol was approved by the Human Research Ethics Committee of the Delft University of Technology and the Agency for Promotion, Investment, and One-Stop Licensing Service at the district level. Participation was voluntary, and we obtained informed consent from all respondents.

### 2.2. Contextual Determinant Variables of Household Hygiene Conditions

We used 11 variables as determinants of household hygiene conditions. These variables were found significantly related or often measured to determine WASH-related behaviors or practices in developing countries, e.g., HWTS practices, sanitation, or handwashing behaviors: (1) wealth [37,38], (2) access to water [36,39], (3) household time allocation or spare time [39,40], (4) local beliefs [36,41,42], (5) access to market [43,44], (6) access to mass media or information [45], (7) receiving WASH promotions [45,46], (8) mother’s education [47,48], (9) father’s education [43,49], and (10) the presence of children under five-years-old [47,48,50]. 

The variable “wealth” was measured by household assets and conditions, e.g., the house’s floor, walls, the availability of electricity, a TV, and a car. “Access to water” was measured in five scales: “below 5 min”, “5–15 min”, “16–30 min”, “31–45 min”, and “>45 min”. The “spare time” was measured in hours and was used to measure the mother’s busyness during the day. The assumption is that a busy mother does not have time for household hygiene-related practices. The local belief was coded as following Marapu or not, i.e., beliefs other than Marapu, e.g., Christianity, Catholicism, and Islam, were coded as “not” a local or indigenous belief. This categorization was made because a previous study found that there is a significant relationship between indigenous beliefs and the HWT practices [36]. “Access to market” was coded as either “difficult access” or “easy access” based on the relative distance to the city center, i.e., Waingapu (Figure 1). The variable “access to mass media” was measured by asking the frequency at which TV was watching in a day using five frequency scales (“almost never”, i.e., score “1”, to “very often”, i.e., score “5”). For the variable “receiving WASH promotions”, the answers were “yes” or “no”, and there was no specific time frame asked for this question, e.g., promotions could have been received in the past month, past year, or even longer. The education of the mother and father were measured by year. Finally, the variable “have children under 5 years old” was coded as “yes” or “no”. 

### 2.3. Hygiene-Related Variables

**Figure 2:**
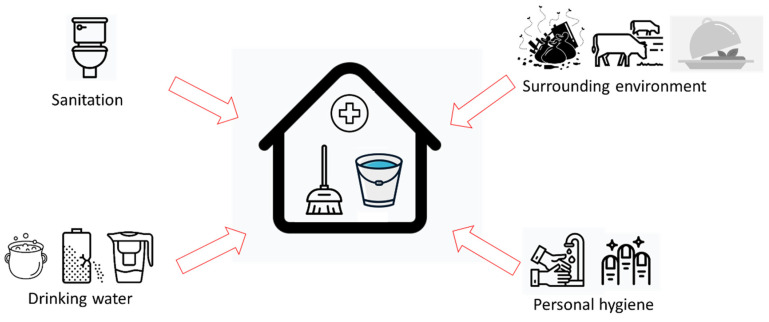
The conceptual model of four clusters of the determinants of household hygiene.

There are 16 hygiene-related variables used in this study (Table 1). As mentioned in the introduction, these variables were selected based on previous household hygiene studies [5,11,12,13,14]. This study categorized those variables into four main clusters of household hygiene: sanitation, surrounding environment, drinking water, and personal hygiene (Figure 2). 

A variable toilet facility is related to sanitation. There are six variables related to the surrounding environment: “livestock nearby”, “floor types”, “floor cleanliness”, “feces around”, “garbage around”, “flies around”, and “food storage”. The next cluster is drinking water, which consists of variables measuring the water storage conditions (cover, crack, placement, and cleanliness), whether they practice household water treatment or not, and this was complemented with drinking water quality, i.e., whether *E. coli* was detected or not. Finally, the last cluster concerns personal hygiene, i.e., related to fingers, which comprises the presence of handwashing facilities and the condition of respondent’s nails. This study assumes that those variables represent general household hygiene conditions. 

### 2.4. Data Analysis

Due to missing data in some respondents, only data from 278 respondents with complete questionnaires were analyzed in this study (85% of the total data). The wealth index was created using the principal component analysis of the household assets and conditions. The first principal component was assumed to represent the relative wealth index of the households [51]. 

To allow comparison between variables, we standardized the scores from “0” (worst condition) to “2” (best condition). For example, in the variable “floor cleanliness”, which has three scales, the value “0” means “dirty”, “1” means “quite dirty”, and “2” means “clean”. For the variables with two scales, i.e., binary variables (“0” or “1”), they were recoded into “0” and “2”. For example, in the variable “practice HWT”, the value “0” means “do not treat drinking water” and “2” means “treat drinking water”. 

In this study, all of these variables were assumed to have equal weight on general household hygiene conditions. The scores were summed to achieve the composite value of general household hygiene conditions, as implied by the sanitary inspection form [9]. This means that the worst possible household hygiene conditions have a score of “0” and that the best conditions have a score of “32”. We then divided the scores into 3 levels to categorize the hygiene level of households: scores 0–10 as “poor hygiene”, scores 11–21 as “moderate hygiene”, and scores 22–32 as “good hygiene”. 

Bivariate nonparametric Chi-squared (X^2^) tests to assess potential relationships between hygiene-related variables were also conducted. The Bonferroni adjustment of the *p*-value was applied to reduce the false-positive result error in the Chi-squared tests. The adjusted Bonferroni *p*-value was 0.0035 (0.05/14). Finally, the forced-entry linear regression analysis, i.e., all independent variables were entered simultaneously into the model, using 10 potential contextual determinants as independent variables on the composite of the value of general household hygiene conditions was conducted. All statistical analyses were conducted using IBM SPSS Statistics 25. 

## 3. Results

### 3.1. Descriptive Statistics

**Figure 3:**
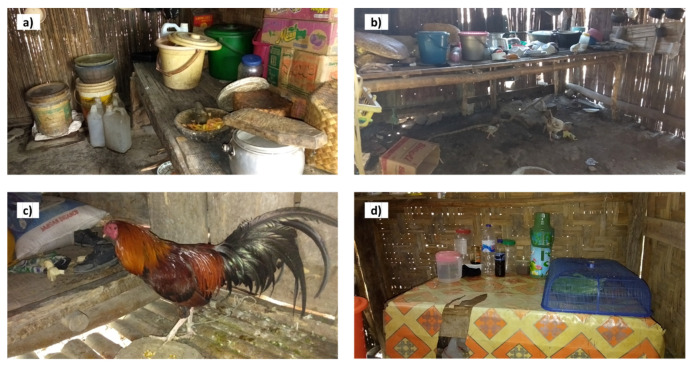
Examples of observable household hygiene conditions in the study area: (**a**) drinking water is stored in a jerry can or a bucket; (**b**) earthen or not permanent kitchen floor and chicken inside the kitchen; (**c**) dirty bamboo kitchen floor with chicken’s feces; (**d**) food is stored inside of the food cover.

Most of the respondents were a mother (84.5%), while others were the father or household head. The proportion of households with and without children under the age of 5 was almost equal, i.e., 47.8% and 52.2%, respectively. The mean schooling time of the mother was 7.7 years (SD = 3.6), while that of the household head or father was 7.4 (SD = 3.6). About one-fourth of the respondents (26.3%) practiced the local belief “Marapu”. The mean spare time of the respondents was 2.9 h in a day (SD = 2.1). In terms of the accessibility of the location of the respondent’s house, about 54% of the respondents were located in an area where it was relatively difficult to access the market. The majority of the respondents had a non-permanent wall (87.8%), e.g., bamboo or wood, and a non-permanent floor (72.3%), e.g., bamboo, earthen, or compacted soil, while only 7.9% had a non-permanent roof, e.g., straw. Having livestock was common, i.e., 89.9% of respondents had at least one pig, and 25.5% had a cow(s). A total of 78.8% of households were observed to cover their food. Figure 3 shows examples of the house kitchen conditions in the study area. 

Thirty-three percent of the respondents drew water from the tap, which was either a private (16.2%) or a public tap (16.5%), while the majority (57.0%) relied on surface water, e.g., a shallow dug well, river, or spring, and 10.1% bought commercial water, e.g., from a refill water station or water truck. More than half of the respondents (51.8%) had a water source that at a close enough distance that it could be reached within a five-minute walk per trip, while 21.5% of the respondents needed to make a walking round trip that was more than a half an hour to acquire water. Open defecation was still practiced by 31.3% of the respondents. Handwashing facilities with water and soap were only available in 39.6% of total households. Only 59.7% of respondents reported that they treat their water. About 87.4% of the respondents mentioned that they have received or participated in promotional WASH activities. 

### 3.2. Hygiene Conditions and the Determinants 

The mean of the composite score was 19.8 (SD = 4.8, range values = 4–31) out of the highest possible value of 32. The households were then categorized into three categories based on their scores, i.e., poor hygiene to good hygiene (see Section 2.4). The percentages of households categorized as “poor hygiene”, “moderate hygiene”, and “good hygiene” were 4.0%, 57.2%, and 38.8%, respectively. 

**Figure 4:**
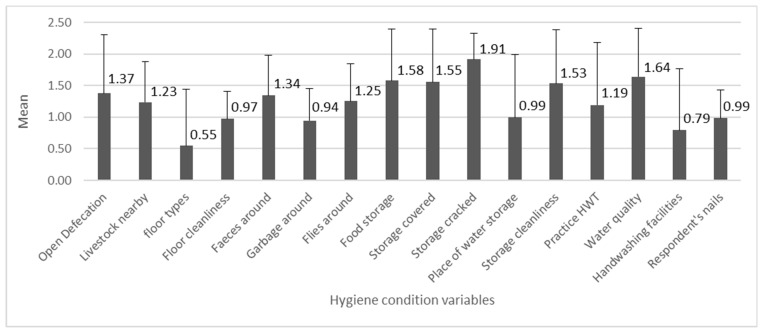
The numbers indicate mean values of hygiene variables (*n* = 278, min = 0, max = 2), with the standard deviation bars.

The mean values of the 16 variables related to the hygiene conditions are shown in Figure 4. The individual variable scores were the lowest on average for “floor types”, followed by “handwashing facilities” (with water and soap), and “garbage around”, and they were the highest for “storage cracked” followed by “water quality” and “food storage”.

The relationship tests between 15 hygiene variables are shown in Table 2. The relationships between the first six variables are related to the surrounding environment, i.e., from “livestock nearby” to “flies around”, were in a positive direction, i.e., the better the condition in one variable, the better the condition in other variables were. For example, no or few livestock nearby the house was associated with fewer flies around. These five variables were also significantly related to the house having no permanent floor. Furthermore, the relationship tests indicate that the poor conditions of these first five variables were associated with having no food cover, i.e., people did not cover their food even though the surrounding environment was not hygienic or clean. 

The relationships between “handwashing facilities” and “respondent’s nails” with “livestock nearby”, “floor cleanliness”, “feces around”, and “garbage around” were in a positive direction. Households who practiced HWT tended to cover their water storage. Furthermore, there was no variable related to “water quality”, i.e., detected *E. coli* in the drinking water, “storage cleanliness”, and “storage cracked”. 

The regression analysis indicated that significant determinants were “wealth” and “access to water” (*p*-value < 0.05; Table 3). The wealthier the households were, the better the household hygiene conditions were, i.e., a positive regression coefficient. On the other hand, difficult access to water was associated with lower hygiene conditions. 

## 4. Discussion

The mean values of composite hygiene and household categorization indicate that most households in the study area did not practice proper hygiene practices in their houses. The majority of the respondents were categorized as having “moderate hygiene”, i.e., 57.2%. There are many potential sources of contamination at the household level and these put them at risk for the spread of pathogens in their houses. The results indicate that the main contamination pathways were through the environment, i.e., non-permanent or earthen floor and garbage, as well as personal hygiene, i.e., handwashing. These findings are in line with other studies that found that indigenous communities often lag behind in terms of WASH facilities or practices [52,53,54]

The risk of pathogen transmission through a non-permanent floor cannot be overlooked, considering that 72.3% of the respondents had a non-permanent floor. Moreover, the non-permanent household floor was associated with a dirty floor or the presence of garbage and flies around. Previous studies indicated that an earthen floor possesses a higher chance of pathogen transmission in Bangladesh, Kenya, and Mozambique [55,56]. This suggests the need to improve the condition of the floors in the houses in the study area. However, low-income households may not be able to afford a permanent floor. In this case, they need a subsidy from the village office. This kind of subsidy, i.e., one for repairing or improving house conditions, is common in the study area but only reaches a few poor houses per year. 

One of the key barriers to prevent contamination and infection is handwashing [57]. However, it seems that people in the study area do not value handwashing as being important. Moreover, the mean value of the variable “respondent’s nails” was also low, indicating that people in the study area were less aware of the importance of personal hygiene. A previous study stated that “general hygiene practice was correlated with commitment to hygiene, indicating a strong association to psychosocial determinants” [12]. Future studies conducted in this area should then investigate the key psychosocial determinants of handwashing that can be targeted in health promotion activities.

There were significant relationships between the six variables related to the surrounding environment, i.e., floor types, floor cleanliness, and presence of livestock, feces, garbage, and flies around. The presence of livestock and allowing them roam freely could be the reason why there are a lot of feces and flies around the house as well as dirty floors. A previous study indicated that fecal contamination in a house is associated with the presence of livestock [14,58,59]. Another study found that the presence of livestock is associated with diarrhea and malnutrition in children [60]. Additionally, considering the fact that East Sumba has one of the highest prevalences of stunting in Indonesia [32], corralling livestock separately from the house’s main areas should be conducted to minimize direct or indirect fecal contamination from livestock [14]. A previous study in Bangladesh found that corralling livestock improves the drinking water quality and reduces cases of diarrhea in children [61]. 

However, interventions related to livestock should also consider the cultural aspect of livestock in the Sumbanese culture. That is because livestock is a symbol of social status [62]. Moreover, some locals said that they prefer to keep their livestock as close as possible to the house to avoid livestock theft. All of these aspects should be considered in the intervention strategies. Furthermore, this study also emphasizes that culture should be taken into account in WASH programs in indigenous communities, i.e., they should avoid altering the local culture [53,63]. 

Furthermore, Figure 3 implies that there is not much difference regarding earthen floors with or without animal feces. This may explain the relationships between not having a permanent or earthen floor and those five variables. People, i.e., those who have an earthen floor, may keep their current unhygienic practices because they have become used to their homes looking dirty, as this may have been the case for a long period of time. 

The results imply that access to water is critical to having proper hygiene practices at home. A previous HWT study in this area stated that “households who need more time to collect water perceived lower levels of ability and self-regulation to operate HWT technologies” [36], which may apply to the context of hygiene practices. Some locals also said that they stopped using a toilet, i.e., they went back to open defecation, because sufficient water is unavailable. Moreover, the health effects of hygiene practices depend on having access to a sufficient water supply [3]. The lack of water in East Sumba is a result of complex environmental conditions. The mean annual rainfall in the study area is about 830 mm/year [30], which is below the mean annual rainfall in the country, i.e., about 2700 mm/year [64]. Moreover, the soil structure complicates people having access to groundwater [63]. The national and local authorities, i.e., village office and municipality, should then focus on delivering water to areas in need. Special attention should be given to the drought period in April–October to ensure that people still have an adequate amount of water to perform proper hygiene behaviors, e.g., delivering water by water trucks during the drought period. Building or subsidizing a rainwater harvesting tank in which to store water during the rainy season could also be another solution. 

The significant and positive influence of wealth on hygiene conditions is consistent with the literature [37,38]. This could be because having more money allows people to purchase items and practice proper hygiene practices. This finding also indicates that low-income households are more vulnerable to contracting diseases due to improper hygiene conditions and practices. Considering the economic conditions of households in the study area, low-cost interventions should be the short-term priority, e.g., regularly cleaning the house of garbage and animal feces as well as cleaning the nails. Furthermore, high-cost interventions, e.g., water provision, a latrine, or a permanent-concrete house floor, can be conducted using a turn-based system using a subsidy from the village office, i.e., these high-cost interventions can be a long-term program. 

This study shows that by including the contextual determinants of hygiene conditions in the analysis, we could design more specific types of intervention. For example, we could urge the target group to engage in handwashing activities regularly, but without analyzing the contextual determinants, we would not have been able to know that one of the possible reasons for irregular handwashing is because there is not a sufficient amount of water, i.e., water needs to be provided first before the suggestion to increase handwashing is given. Analyzing these contextual determinants may help us to understand the “root causes” of health-related behaviors because without tackling the problem at the “root”, we may not be able to transform people’s unhealthy behaviors. 

This study also indicates that rural and less developed areas in Indonesia, i.e., where the majority of households have low income and education levels, need a special focus on hygiene interventions. Multiple stakeholders at the district level, e.g., district health agency, social agency, NGOs, etc., should work together to tackle this issue. Health posts in the sub-district level and in the pre- and postnatal healthcare information (called “Posyandu” in Bahasa) in the village level can be the hygiene promotion center. Moreover, based on our discussion with some of the stakeholders in that area, support from higher-level institutions, e.g., agencies at the provincial or national level, is urgently needed, especially regarding high-cost interventions, e.g., water supply [63]. 

This study has some limitations. First, even though enumerator training and the pilot study were conducted before the real data collection period, there is the potential for inconsistency regarding the assessment of the hygiene variables among the different enumerators [65]. Second, we relied on self-reported answers o regarding water treatment which may be a source of bias, as found in another HWT in Cambodia [66]. Third, the water quality analyses were only conducted at a single time point and only presence-absence (P/A) tests were conducted. Thus, temporal variation in the water quality was not recorded, and P/A test may not fully capture the actual water quality. Fourth, seasonal hygiene conditions, i.e., those in the rainy and dry seasons, should also be conducted since access to water is abundant in the rainy season but limited in the dry season. Fifth, future studies should also investigate the relationship between general household conditions and water-related diseases in children, e.g., diarrhea and malnutrition. Sixth, the hygiene variables used in this study may not contribute equally to the general household hygiene. Future studies should investigate this so that we can have a better proximation of general household hygiene conditions. Moreover, a behavioral study should be conducted to understand the underlying perceptions or psychological factors behind current hygiene conditions, including the investigation of other contextual factors that may influence hygiene conditions, e.g., policy, regulations, WASH-related institutional performance, etc. These findings could help to design the behavioral change interventions that are needed in the study area. 

## 5. Conclusions

This study examines the general household hygiene conditions in less developed rural areas in Indonesia. In general, the hygiene conditions were at a “moderate” level, indicating that people in the study area were at risk for poor hygiene-related diseases. Two important contamination pathways were the surrounding environment, i.e., a non-permanent floor and the presence of garbage, and personal hygiene., i.e., handwashing. There is a need to improve non-permanent floors to permanent floors, but a subsidy is needed for low-income households. The presence of livestock roaming freely in the yard is one of the reasons for the dirty surrounding environment. However, the cultural aspect of livestock ownership should be taken into account in intervention strategies. Intervention should also target handwashing behaviors and nail cleaning to prevent the spread of contaminants through the fingers. Having access to water positively influences hygiene conditions. This is a serious challenge in this area because people regularly face water scarcity 7–8 months a year. Wealth also significantly influences household hygiene conditions, indicating that poor households should be the main target group WASH interventions. Low-cost interventions can be conducted in terms of immediate interventions, e.g., regularly cleaning the house of garbage and cleaning one’s nails. Finally, this study can be seen as a snapshot of the household hygiene conditions in less developed and rural areas in Indonesia and as a trigger to improve household health in those areas. 

## Figures and Tables

**Table 1:** Household hygiene-related variables used in this study.

Variables	Questions	Scale *
Open Defecation	What types of toilets do you have?	2 (Open defecation or not)
Livestock nearby	Is there livestock around the house?	3 (Many–few)
Floor types	What type of floor does the main house have?	2 (Permanent or not)
Floor cleanliness	How is the cleanliness of the house floor?	3 (Dirty–clean)
Feces around	Is there human or animal feces in the yard (or even inside the house)?	3 (Many–few)
Garbage around	Is there garbage around the house?	3 (Many–few)
Flies around	Can you see flies around the water storage container?	3 (Many–few)
Food storage	How do you store cooked food?	2 (with or without cover)
Storage covered	Is the water storage being covered (at that time)?	2 (Yes or not)
Storage cracked	Is the container cracked?	2 (Yes or not)
Place of water storage	When not in use, is the storage container kept in a place where it may become contaminated?	2 (Yes or not)
Storage cleanliness	Is the inside of the container clean?	2 (Dirty or clean)
Practice household water treatment (HWT)	Is the drinking water treated?	2 (Yes or not)
E. coli detected	Data were from quality testing the drinking water	2 (Yes or not)
Handwashing facilities	What kind of handwashing facilities does the household have?	2 (With water and soap or not)
Respondent’s nails	How clean are the respondent nails?	3 (Dirty–clean)

* ”2” indicates binary variables, i.e., “0” and “1”, and “3” means that the variable is measured in three levels, i.e., 0, 1, and 2.

**Table 2:** Relationship between 15 hygiene-related variables. Green cells indicate significant relationships (*p* ≤ 0.0035 after the Bonferroni adjustment).

	Open Defecation	Livestock Nearby	Floor Types	Floor Cleanliness	Faeces Around	Garbage Around	Flies Around	Food Storage	Storage Covered	Storage Cracked	Place of Water Storage	Storage Cleanliness	Practice HWT	Water Quality	Handwashing Facilities	Respondent’s Nails
Open Defecation																
Livestock nearby																
Floor types																
Floor cleanliness																
Faeces around																
Garbage around																
Flies around																
Food storage																
Storage covered																
Storage cracked																
Place of water storage																
Storage cleanliness																
Practice HWT																
Water quality																
Handwashing facilities																
Respondent’s nails																

**Table 3:** Regression analysis of determinants of household hygiene conditions.

Determinant Variables	B	SE	β	*p* Value	LB	UB
Constant	20.22	1.31		0.00	17.65	22.80
Wealth	1.03	0.38	0.23	0.01	0.29	1.78
Access to water	−0.43	0.20	−0.14	0.04	−0.83	−0.03
Household time allocation	0.06	0.13	0.03	0.63	−0.19	0.31
Local beliefs	−0.87	0.65	−0.09	0.19	−2.16	0.42
Access to market	0.93	0.53	0.10	0.08	−0.11	1.96
Access to mass media	0.15	0.24	0.05	0.52	−0.32	0.62
Receiving WASH promotions	−0.11	0.78	−0.01	0.89	−1.64	1.42
Mother’s education	−0.10	0.10	−0.08	0.32	−0.28	0.09
Father’s education	0.01	0.09	0.01	0.88	−0.17	0.20
Having children under 5-years-old	0.00	0.52	0.00	1.00	−1.02	1.02

*n* = 278; Adj. R^2^ = 0.111.

## Data Availability

The data presented in this study are openly available in the repository Narcis at https://doi.org/10.4121/uuid:9fb7a522-06a1-457c-9c69-3b2529c38d92.

## References

[1] Wolf J., Hunter P.R., Freeman M.C., Cumming O., Clasen T., Bartram J., Higgins J.P.T., Johnston R., Medlicott K., Boisson S. (2018). Impact of drinking water, sanitation and handwashing with soap on childhood diarrhoeal disease: Updated meta-analysis and meta-regression. Trop. Med. Int. Health.

[2] Freeman M.C., Stocks M.E., Cumming O., Jeandron A., Higgins J., Wolf J., Prüss-Ustün A., Bonjour S., Hunter P., Fewtrell L. (2014). Systematic review: Hygiene and health: Systematic review of handwashing practices worldwide and update of health effects. Trop. Med. Int. Health.

[3] Cairncross S., Hunt C., Boisson S., Bostoen K., Curtis V., Fung I.C.-H., Schmidt W.-P. (2010). Water, sanitation and hygiene for the prevention of diarrhoea. Int. J. Epidemiol..

[4] Cumming O., Cairncross S. (2016). Can water, sanitation and hygiene help eliminate stunting? Current evidence and policy implications. Matern. Child Nutr..

[5] Daniel D., Iswarani W.P., Pande S., Rietveld L. (2020). A Bayesian Belief Network model to link sanitary inspection data to drinking water quality in a medium resource setting in rural Indonesia. Sci. Rep..

[6] Gundry S., Wright J., Conroy R. (2004). A systematic review of the health outcomes related to household water quality in developing countries. J. Water Health.

[7] WHO (2012). Water Safety Planning for Small Community Water Supplies: Step-by-Step Risk Management Guidance for Drinking-Water Supplies in Small Communities.

[8] Ercumen A., Mertens A., Arnold B.F., Benjamin-Chung J., Hubbard A.E., Ahmed M.A., Kabir M.H., Khalil M.R., Kumar A., Rahman S. (2018). Effects of Single and Combined Water, Sanitation and Handwashing Interventions on Fecal Contamination in the Domestic Environment: A Cluster-Randomized Controlled Trial in Rural Bangladesh. Environ. Sci. Technol..

[9] Kelly E.R., Cronk R., Kumpel E., Howard G., Bartram J. (2020). How we assess water safety: A critical review of sanitary inspection and water quality analysis. Sci. Total Environ..

[10] WHO (1997). Surveillance and Control of Community Supplies, Guidelines for Drinking-Water Quality.

[11] Daniel D., Gaicugi J., King R., Marks S.J., Ferrero G. (2020). Combining Sanitary Inspection and Water Quality Data in Western Uganda: Lessons Learned from a Field Trial of Original and Revised Sanitary Inspection Forms. Resources.

[12] Sonego I.L., Mosler H.-J. (2016). Spot-checks to measure general hygiene practice. Int. J. Environ. Health Res..

[13] Agustina R., Sari T.P., Satroamidjojo S., Bovee-Oudenhoven I.M., Feskens E.J., Kok F.J. (2013). Association of food-hygiene practices and diarrhea prevalence among Indonesian young children from low socioeconomic urban areas. BMC Public Health.

[14] Daniel D., Diener A., Van De Vossenberg J., Bhatta M., Marks S.J. (2020). Assessing Drinking Water Quality at the Point of Collection and within Household Storage Containers in the Hilly Rural Areas of Mid and Far-Western Nepal. Int. J. Environ. Res. Public Health.

[15] Marjadi B., McLaws M.-L. (2010). Hand hygiene in rural Indonesian healthcare workers: Barriers beyond sinks, hand rubs and in-service training. J. Hosp. Infect..

[16] Hirai M., Graham J.P., Mattson K.D., Kelsey A., Mukherji S., Cronin A.A. (2016). Exploring Determinants of Handwashing with Soap in Indonesia: A Quantitative Analysis. Int. J. Environ. Res. Public Health.

[17] Dwipayanti N.M.U., Lubis D.S., Harjana N.P.A. (2021). Public Perception and Hand Hygiene Behavior during COVID-19 Pandemic in Indonesia. Front. Public Health.

[18] Akombi B.J., Agho K.E., Hall J.J., Wali N., Renzaho A., Merom D. (2017). Stunting, wasting and underweight in Sub-Saharan Africa: A systematic review. Int. J. Environ. Res. Public Health.

[19] Torlesse H., Cronin A.A., Sebayang S.K., Nandy R. (2016). Determinants of stunting in Indonesian children: Evidence from a cross-sectional survey indicate a prominent role for the water, sanitation and hygiene sector in stunting reduction. BMC Public Health.

[20] Beal T., Tumilowicz A., Sutrisna A., Izwardy D., Neufeld L.M. (2018). A review of child stunting determinants in Indonesia. Matern. Child Nutr..

[21] Rachmi C.N., Agho K.E., Li M., Baur L. (2016). Stunting, Underweight and Overweight in Children Aged 2.0–4.9 Years in Indonesia: Prevalence Trends and Associated Risk Factors. PLoS ONE.

[22] Irianti S., Prasetyoputra P. (2021). Rural–Urban Disparities in Access to Improved Sanitation in Indonesia: A Decomposition Approach. SAGE Open.

[23] Irianti S., Prasetyoputra P., Sasimartoyo T.P. (2016). Determinants of household drinking-water source in Indonesia: An analysis of the 2007 Indonesian family life survey. Cogent Med..

[24] Patunru A.A. (2015). Access to Safe Drinking Water and Sanitation in Indonesia. Asia Pac. Policy Stud..

[25] Braveman P., Gottlieb L. (2002). The Social Determinants of Health: It’s Time to Consider the Causes of the Causes. Public Health Rep..

[26] Mosler H., Contzen N. (2016). Systematic Behavior Change in Water Sanitation and Hygiene. A Practical Guide Using the RANAS Approach. Version 1.1. https://research.rug.nl/en/publications/systematic-behavior-change-in-water-sanitation-and-hygiene-a-prac.

[27] Fitzpatrick C., Engels D. (2016). Leaving no one behind: A neglected tropical disease indicator and tracers for the Sustainable Development Goals: Box 1. Int. Health.

[28] Statistics of Sumba Timur Regency (2018). Persentase Rumah Tangga Menurut Fasilitas Tempat Buang Air Besar (Persen), 2015–2017. Statistics of Sumba Timur Regency..

[29] BPS Statistics of East Sumba Regency (2018). Persentase Rumah Tangga Menurut Sumber Air Utama yang Digunakan Untuk Minum di Kabupaten Sumba Timur, 2015–2017. Statistics of Sumba Timur Regency..

[30] Messakh J.J., Moy D.L., Mojo D., Maliti Y. (2018). The linkage between household water consumption and rainfall in the semi-arid region of East Nusa Tenggara, Indonesia. IOP Conf. Ser. Earth Environ. Sci..

[31] Vel J.A.C., Makambombu S. (2010). Access to Agrarian Justice in Sumba, Eastern Indonesia. Law Soc. Justice Glob. Dev..

[32] Picauly I., Toy S.M. (2013). Analisis Determinan dan Pengaruh Stunting Terhadap Prestasi Belajar Anak Sekolah di Kupang dan Sumba Timur, Ntt. J. Gizi dan Pangan.

[33] Sungkar S., Pohan A.P.N., Ramadani A., Albar N., Azizah F., Nugraha A.R.A., Wiria A.E. (2015). Heavy burden of intestinal parasite infections in Kalena Rongo village, a rural area in South West Sumba, eastern part of Indonesia: A cross sectional study. BMC Public Health.

[34] BPS Statistics of East Timur Regency (2019). Sumba Timur in Figures 2019. http://sbdkab.go.id/sbdku/Kabupaten%20Sumba%20Barat%20Daya%20Dalam%20Angka%202019%20-%20Kominfo.pdf.

[35] Daniel D., Pande S., Rietveld L. (2020). The effect of socio-economic characteristics on the use of household water treatment via psychosocial factors: A mediation analysis. Hydrol. Sci. J..

[36] Daniel D., Pande S., Rietveld L. (2021). Socio-Economic and Psychological Determinants for Household Water Treatment Practices in Indigenous–Rural Indonesia. Front. Water.

[37] Munamati M., Nhapi I., Misi S. (2016). Exploring the determinants of sanitation success in Sub-Saharan Africa. Water Res..

[38] Roma E., Bond T., Jeffrey P. (2014). Factors involved in sustained use of point-of-use water disinfection methods: A field study from Flores Island, Indonesia. J. Water Health.

[39] Figueroa M., Kincaid D. (2010). Social, Cultural and Behavioral Correlates of Household Water Treatment and Storage. Cent Publ HCI 2010-1 Heal Commun Insights, Balt Johns Hopkins Bloom Sch Public Heal Cent Commun Programs. http://ccp.jhu.edu/wp-content/uploads/Household-Water-Treatment-and-Storage-2010.pdf.

[40] Chittleborough C.R., Nicholson A.L., Basker E., Bell S.L., Campbell R. (2012). Factors influencing hand washing behaviour in primary schools: Process evaluation within a randomized controlled trial. Health Educ. Res..

[41] Behailu B.M., Pietilä P.E., Katko T. (2016). Indigenous Practices of Water Management for Sustainable Services. SAGE Open.

[42] Waterworth P., Pescud M., Braham R., Dimmock J., Rosenberg M. (2015). Factors Influencing the Health Behaviour of Indigenous Australians: Perspectives from Support People. PLoS ONE.

[43] Dubois A.E., Crump J.A., Keswick B.H., Slutsker L., Quick R.E., Vulule J.M., Luby S.P. (2010). Determinants of Use of Household-level Water Chlorination Products in Rural Kenya, 2003–2005. Int. J. Environ. Res. Public Health.

[44] Goldman N., Pebley A.R., Beckett M. (2001). Diffusion of ideas about personal hygiene and contamination in poor countries: Evidence from Guatemala. Soc. Sci. Med..

[45] George C.M., Jung D.S., Saif-Ur-Rahman K.M., Monira S., Sack D.A., Rashid M.U., Toslim M., Mustafiz M., Rahman Z., Bhuvian S.I. (2016). Sustained Uptake of a Hospital-Based Handwashing with Soap and Water Treatment Intervention (Cholera-Hospital-Based Intervention for 7 Days [CHoBI7]): A Randomized Controlled Trial. Am. J. Trop. Med. Hyg..

[46] Mosler H.-J., Kraemer S., Johnston R. (2013). Achieving long-term use of solar water disinfection in Zimbabwe. Public Health.

[47] Freeman M.C., Trinies V., Boisson S., Mak G., Clasen T. (2012). Promoting Household Water Treatment through Women’s Self Help Groups in Rural India: Assessing Impact on Drinking Water Quality and Equity. PLoS ONE.

[48] Totouom A.L.F., Sikod F., Abba I. (2012). Household Choice of Purifying Drinking Water in Cameroon. Environ. Manag. Sustain. Dev..

[49] Nauges C., Van Den Berg C. (2009). Perception of Health Risk and Averting Behavior: An Analysis of Household Water Consumption in Southwest Sri Lanka. Toulouse Sch. Econ. Work Pap..

[50] Christen A., Pacheco G.D., Hattendorf J., Arnold B.F., Cevallos M., Indergand S., Colford J.M., Mäusezahl D. (2011). Factors associated with compliance among users of solar water disinfection in rural Bolivia. BMC Public Health.

[51] Houweling T.A., Kunst A., MacKenbach J.P. (2003). Measuring health inequality among children in developing countries: Does the choice of the indicator of economic status matter?. Int. J. Equity Health.

[52] Hall N.L. (2019). Challenges of WASH in remote Australian Indigenous communities. J. Water Sanit. Hyg. Dev..

[53] Jiménez A., Cortobius M., Kjellén M. (2014). Water, sanitation and hygiene and indigenous peoples: A review of the literature. Water Int..

[54] Latchmore T., Schuster-Wallace C.J., Longboat D.R., Dickson-Anderson S., Majury A. (2018). Critical elements for local Indigenous water security in Canada: A narrative review. J. Water Health.

[55] Benjamin-Chung J., Crider Y.S., Mertens A., Ercumen A., Pickering A.J., Lin A., Steinbaum L., Swarthout J., Rahman M., Parvez S.M. (2021). Household finished flooring and soil-transmitted helminth and Giardia infections among children in rural Bangladesh and Kenya: A prospective cohort study. Lancet Glob. Health.

[56] Holcomb D., Knee J., Sumner T., Adriano Z., de Bruijn E., Nalá R., Cumming O., Brown J., Stewart J.R. (2020). Human fecal contamination of water, soil, and surfaces in households sharing poor-quality sanitation facilities in Maputo, Mozambique. Int. J. Hyg. Environ. Health.

[57] Mathur P. (2011). Hand hygiene: Back to the basics of infection control. Indian J. Med. Res..

[58] Ercumen A., Pickering A.J., Kwong L.H., Arnold B.F., Parvez S.M., Alam M., Sen D., Islam S., Kullmann C., Chase C. (2017). Animal Feces Contribute to Domestic Fecal Contamination: Evidence from E. coli Measured in Water, Hands, Food, Flies, and Soil in Bangladesh. Environ. Sci. Technol..

[59] Wardrop N.A., Hill A.G., Dzodzomenyo M., Aryeetey G., Wright J.A. (2018). Livestock ownership and microbial contamination of drinking-water: Evidence from nationally representative household surveys in Ghana, Nepal and Bangladesh. Int. J. Hyg. Environ. Health.

[60] Penakalapati G., Swarthout J., Delahoy M.J., McAliley L., Wodnik B., Levy K., Freeman M.C. (2017). Exposure to Animal Feces and Human Health: A Systematic Review and Proposed Research Priorities. Environ. Sci. Technol..

[61] Ercumen A., Naser A.M., Unicomb L., Arnold B., Colford J.M., Luby S. (2015). Effects of Source- versus Household Contamination of Tubewell Water on Child Diarrhea in Rural Bangladesh: A Randomized Controlled Trial. PLoS ONE.

[62] Bamualim A., Livestock Production and Fire Management in East Nusa Tenggara Fire and Sustainable Agricultural and Forestry Development in Eastern Indonesia and Northern Australia. Proceedings of an International Workshop Held at Northern Territory University, Darwin, Australia, 13–15 April 1999; pp. 69–72. https://png-data.sprep.org/system/files/Fire%20and%20Sustainable%20Agricultural%20and%20Forestry%20Development%20in%20Eastern%20Indonesia%20and%20Northern%20Australia.pdf.

[63] Daniel D., Djohan D., Machairas I., Pande S., Arifin A., Al Djono T.P., Rietveld L. (2021). Financial, institutional, environmental, technical, and social (FIETS) aspects of water, sanitation, and hygiene conditions in indigenous—rural Indonesia. BMC Public Health.

[64] The World Bank (2014). Average Precipitation in Depth (mm per Year)—Indonesia.

[65] Okotto-Okotto J., Wanza P., Kwoba E., Yu W., Dzodzomenyo M., Thumbi S.M., da Silva D.T.G., Wright J.A. (2020). An Assessment of Inter-Observer Agreement in Water Source Classification and Sanitary Risk Observations. Expo. Health.

[66] Brown J., Sobsey M.D. (2012). Boiling as Household Water Treatment in Cambodia: A Longitudinal Study of Boiling Practice and Microbiological Effectiveness. Am. J. Trop. Med. Hyg..

